# Maker communities and the COVID-19 pandemic: a longitudinal analysis of Thingiverse’s response to supply shortages

**DOI:** 10.1098/rsos.230790

**Published:** 2023-09-27

**Authors:** H. Felton, O. Schiffmann, M. Goudswaard, J. Gopsill, C. Snider, R. Real, A. McClenaghan, B. Hicks

**Affiliations:** Design Manufacturing Futures Laboratory, Department of Mechanical Engineering, University of Bristol, Bristol BS8 1TR, UK

**Keywords:** three-dimensional printing, COVID-19, data analysis

## Abstract

The COVID-19 pandemic profoundly affected various aspects of daily life, particularly the supply and demand of essential goods, resulting in critical shortages. This included personal protective equipment for medical professionals and the general public. To address these shortages, online ‘maker communities’ emerged, aiming to develop and locally manufacture critical products. While some organized efforts existed, the majority of initiatives originated from individuals and groups on platforms like Thingiverse. This paper presents a longitudinal analysis of Thingiverse, one of the largest maker community websites, to examine the pandemic’s effects. Our findings reveal a surge in community output during the initial lockdown periods in major contributing nations (primarily those in the Western Hemisphere), followed by a subsequent decline. Additionally, throughout 2020, pandemic-related products dominated uploads and interactions during this period. Based on these observations, we propose recommendations to expedite the community’s ability to support local, national and international responses to future disasters.

## 1. Introduction

Additive manufacturing (AM), commonly known as three-dimensional printing, has transformed the landscape in which parts are made [1]. Although high-value products continue to be made by corporations centrally, lower-value products and highly customized parts are being increasingly produced at a local level using personal machines [2]. This has led to the field known as the democratization of manufacture, where the freedom to manufacture products is in the hand of the people and production is brought to the masses [3–5].

While capable manufacture resources are available locally in the form of three-dimensional printers, the capacity to design for it is more limited. To generate digital models for printing, computer aided design software can be used, however, it is limited in its accessibility owing to the domain-specific expertise required to design with it [6–8]. While some new design methodologies have been developed to enable the democratization of design [9,10],^1^ the principle alternative means to access designs is through the use of design repositories.

These repositories are typically online locations for creators to share their designs, with examples including Thingiverse [11], Youmagine [12] and GrabCAD [13] (among many others). As AM technologies have become more widely used, the popularity (and number) of these design repositories has followed. While they allow creators to share their designs, and potentially earn an income, their greatest value may lie in the deskilling of the design process. This allows a wide range of users access designs such that they are able to manufacture more complex products at a local level.

Stored in these repositories are a wide variety of parts. Uploads range from three-dimensional print tests—such as the popular ‘3D Benchy’ [14]—to miniatures, and from cable holders to microfluidic devices used in academic work [15,16]. Through this wide variety of parts users are able to experiment and fabricate what they deem as useful.

At the start of the COVID-19 pandemic, the World Health Organisation warned of a global shortage of personal protective equipment (PPE), calling on industry to increase manufacturing to help fill this gap [17]. Owing to the flexibility of AM to quickly adapt to the manufacture of new designs, three-dimensional printing saw a global rise [18] in providing rapid solutions to this shortfall—as well as a variety of other problems [19–23]. Often, these initiatives were led by corporations, health bodies and leading AM machine manufacturers. Examples include the production of nasal swabs by Formlabs [24], the manufacture of face shields by Prusa [25] and three-dimensionally printed respirators by a collaboration between Consorci de la Zona Franca (CZFB), HP, Leitat, SEAT, the Consorci Sanitari de Terrassa and the Taulí Hospital in Sabadell.

As a result of the pandemic, many new organizations such as Open Source Medical Supplies [26] formed and joined the efforts of existing maker communities such as FabLabs [27] in developing designs for combatting COVID-19 and its spread. Often starting as local initiatives, the availability of designs online allowed these efforts to spread nationally and internationally. The Prusa face mask was fabricated by groups in the UK, USA, Canada, France and elsewhere, allowing over 200 000 face shields to be produced [28]. These initiatives were able to successfully use the large number of printers available—predicted to be 151 000 in the UK alone [29]—to alleviate some of the supply chain issues. One area not so well documented, however, is how undirected makers used open design repositories during the pandemic response.

To investigate this, we present a detailed study of the dynamics of design repositories during the pandemic focussing specifically on the largest repository—Thingiverse. Starting with a brief characterization of the data to provide a baseline, the authors have then examined the composition of uploads to Thingiverse in 2020—both as a whole and related directly to COVID. The observed behaviours provide some insights into if and how the maker community could be further used in future scenarios where a directed, local and flexible design and manufacturing approach is needed.

## 2. Maker community data

The data were retrieved from Thingiverse using the Thingiverse application programming interface (API). This section reviews this process, presents the form of data received and demonstrates consistency in the data retrieved.

### 2.1. The Thingiverse application programming interface

One of the most popular design repositories at the time of writing was Thingiverse [30], and so was the focus for this work. Thingiverse operates nearly entirely through user contributions and interaction (with very few exceptions where MakerBot—who used to own Thingiverse—have uploaded their own designs). The users of Thingiverse can upload original designs—known as ‘things’—like and comment on other designs, and ‘remix’ other designs; where the original design is modified for a specific use case or to make small adjustments. To be consistent with the terminology used on the Thingiverse repository, users’ designs will hereby be referred to as *things* throughout this work.

To access the Thingiverse repository data the Thingiverse API was used, focusing on the use of the ‘search’ query type for this work. The API is based on a REST architecture and, at the time of data collation, was freely accessible to anyone who registered with Thingiverse as a developer. The API, as well as the client used to access it, can be found at [31,32], respectively.

The data required to complete the analysis within this work was accessed through automated calls using the *requests-futures* module in Python [33], that allowed multiple simultaneous API calls. This work does not intend to go into the detail of this code as there are several ways to retrieve the same data from the API; however, in short, the code required a query to be provided by the user that was then sent to the request-futures module. This module then sent the request to the Thingiverse API, cycling through every page of results for the given query. Often, this was looped over a time period using the datetime module to ensure complete data collection—discussed further in §2.2.

To align the work with the Thingiverse terms and conditions at the time the data were pulled, no copies of the data used have been made available publicly. However, much of these data remain available on the Thingiverse platform. It should be noted, however, that any future work may find that the data pulled from the API is somewhat different to that used in this work as users are able to change their own data and interact with others’. This is expanded upon further within §2.2.

To demonstrate that the data used was consistent with the state of Thingiverse at the time of writing, work was undertaken that investigated the consistency in data retrieved using different methods at the same point in time, as in §2.3.

### 2.2. Data collation

The data that can be pulled using the Thingiverse API is structured using the JSON format. For a given search—the method used within this work to reference the API—the returned JSON provides a breakdown of the total number of hits and details related to these hits, for up to 10 000 things. If a search returns more than 10 000 hits, the 10 000 hits that best match the search term and order provided are returned. To overcome this limitation, in most instances, the queries sent to the API were broken down by date for the time period of interest. This allowed the full dataset to be retrieved.

**Figure 1.**
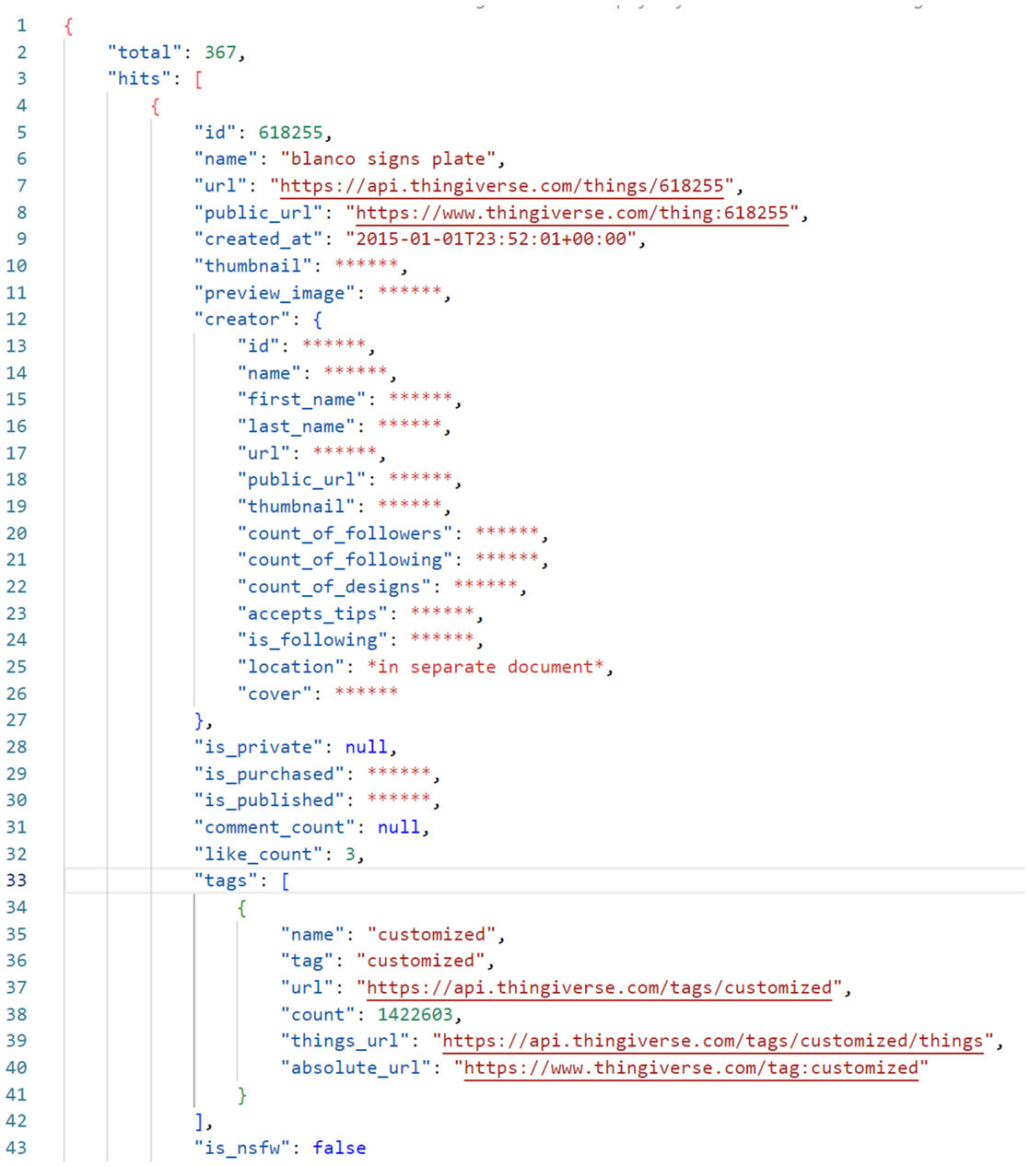
An example hit-organized JSON returned from the search query type by the Thingiverse API. The file will repeat in the same format as shown for every hit. All things that may identify the identity of the creator have been removed and replaced with ‘*****’.

The hit-organized data (figure 1) contain information relating to the thing’s name, identity (ID), creator, number of likes, number of comments and more. This is more fully documented by Thingiverse’s developer documentation [11] but an example is presented in figure 1. Several parts of the data return were deleted to anonymize the output. An anonymized dataset can be found at [34], with permission from Thingiverse. Location data have been limited to the top 100 locations for each query type, and checked to ensure no personal information is included.

The data returned by the API provided a definition for the thing(s) at a single point in time, with some of these data persistent (for example, the ID of the part) and some temporal (for example, the number of likes the thing had). It may be argued that the entirety of the data that defines a thing is temporal—as it may all be deleted by a user. This was found unlikely to be of concern with just 0.27% of the total things uploaded between 1 January 2015 and 31 December 2020 found to have been deleted between March and July 2021.

Based on this information, the authors defined temporal data as data that may be changed through anything other than part deletion. For a search query, as in the example shown, the thing’s only persistent, unique metrics were:

— id,
— created at (date),
— creator id (anonymized).

All other thing properties returned were considered temporal and could change with user interaction. In itself this is unlikely to cause significant issue, and can provide some interesting insights—such as the time period which a thing is typically managed for and interacted with by users. However, owing to the complexity this may have on the conclusions that may be drawn from the work and lack of anonymity of results, the authors used data from 6 July 2021 exclusively using broad search methods. As such, it is generally not possible to reliably retrieve a breakdown of when on-service events happen (i.e. a user likes a design), and so the context around the observations must be considered.

### 2.3. Data validation

To increase confidence that the dataset from Thingiverse was consistent between pull requests when queried at the same time, to avoid issues around temporal data, tests were completed using independent code and calls from separate intellectual property (IP) addresses with different app IDs. These queries, constrained to search methods for consistency with the work presented here, were:

(i) a search for ‘’ (meaning the search term was blank) on 11 March 2016 (randomly generated date), sorted by newest; and
(ii) a search for ‘car’ over July 2019 (again, randomly generated), sorted by most popular.

Both methods used Python commands and the ‘Requests’ module to send GET requests to the Thingiverse API. The number of requests per page and how the code worked through the pagination were not kept consistent. For this reason, there may have been small discrepancies between the returned datasets in temporal data as described in §2.2. A Python script was used to compare the returned files based on the ‘intersection’ method. This read the files line by line and returned any differences in a separate file. No differences were found in the returned data.

Away from the data validation exercise undertaken, it was observed that some of the data had unexpected values. For example, some of the hits returned negative values for likes, despite this not being possible through the Thingiverse interface. For these things, the rest of the data seemed reasonable. Further investigation is presented below to identify what may have been causing this issue.

## 3. Review of data

This section presents and compares the pre-COVID-19 (2015–2019) and COVID-19 (2020) Thingiverse landscapes to one another to try and identify the similarities and differences. This is done over several aspects:

(i) total things;
(ii) types of thing; and
(iii) user interaction with things.

First, however, it was thought necessary to consider where things were being uploaded from to understand where the COVID-19 context should be focused. To do this, the top five countries for upload activity were considered. These countries were found using the ‘location’ field of the Thingiverse user base, with the user of each uploaded part considered (so if the same user uploaded two things, they would be considered twice). Unfortunately, this was a free-text field on Thingiverse, and so entered values varied considerably. To accommodate for this, post-processing of the locations using natural language processing (via Python’s ntk module) was used. This looked for countries, states, counties and cities from publicly available reference lists—taking account of acronyms and alternative formatting. If a match was found, the corresponding United Nations (UN) country was assigned to the user and added to the results list. If no match was found, the entry was removed. This caused a few issues with the developed data:

(i) countries, states (national and international), counties and cities were prioritized in order, with cities also being processed alphabetically by country. As such, locations with the same name in multiple countries may have been incorrectly allocated;
(ii) English language places names were generally used—skewing the list of recognized places in favour of English speaking nations; and
(iii) societal considerations around sharing location could not be considered, which may disproportionately affect some nations, or larger regions, more than others.

For these reasons, the data should not be considered a guarantee of the user base of Thingiverse, but an indication of where uploads have been sourced from. For reference, 196 841 things did not have a given location and 419 provided locations could not be interpreted (owing to encoding errors) for all things uploaded in 2020 (out of a total of 365 799 things). The top five locations for 2015–2019 and 2020 are given in table 1. From these data, it is clear that residents of the USA uploaded more things to Thingiverse in both the reference (2015–2019) and 2020 periods. Interestingly, however, the difference between the number of things uploaded from the USA and other countries was greater for the reference 2015–2019 period than for 2020 (3–5 times more compared to 2–3 times more). This may be owing to interest in uploaded material, users from the USA moving to other platforms, or stagnation in the number of users from the USA relative to the other nations. Still, the number of uploads from the USA was much greater than for any other nation, and thus restrictions associated with COVID-19 that were brought in by the USA will be considered to provide real-world context.

**Table 1.** Top five upload locations for all things in 2015–2019 and 2020.

2015–2019		2020	
country	things (no.)	country	things (no.)
USA	145 599	USA	32 599
Germany	51 611	Germany	15 628
UK	35 414	France	9975
France	35 287	UK	9787
Canada	30 096	Canada	8241

### 3.1. Total things

To investigate how the total number of things has changed over time, data were first collated for all things between 1 January 2015 and 31 December 2019 (inclusive). This provided a 5-year history of how the Thingiverse database had developed and matured. Table 2 presents the yearly increase in things uploaded year-on-year, showing that the site was increasing in popularity over this period. One noteworthy value from table 2 is the year-on-year increase for 2018, just a sixth of the previous year and less than a tenth of that for 2019. It was hypothesized this may have been owing to the rise of alternative repositories or reduced design output owing to saturation of concepts.

**Table 2.** Thingiverse yearly uploads.

year	uploads (no.)	year-on-year change	percentage change
2015	193 717	—	—
2016	241 295	47 578	24.6%
2017	278 267	36 972	15.3%
2018	284 743	6476	2.33%
2019	351 630	66 887	23.5%
2020	365 799	14 169	4.03%

**Figure 2.**
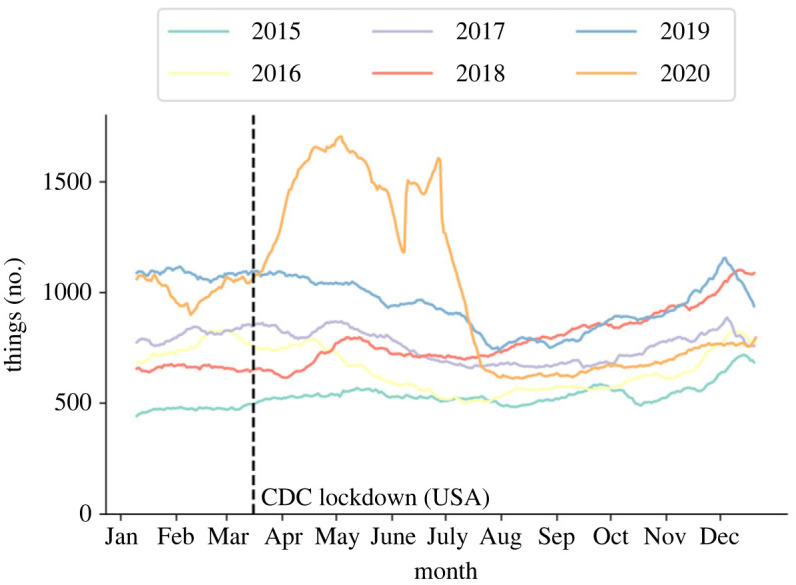
Baseline data for all things uploaded to Thingiverse between 2015 and 2019 along with 2020 data. The vertical line represents when COVID shutdowns were being introduced and enforced in the USA [35]. CDC, Centre for Disease Control.

To further investigate the general increase in uploaded things, and to provide a baseline dataset for the COVID period (2020) to compare to, the daily trend of all things uploaded was plotted, as in figure 2, between 2015 and 2019. These data were averaged over one (2019), three (2017–2019) and five (2015–2019) consecutive years, and time-averaged over 21 days to avoid spikes in uploads and observe general trends. Leap year days were not considered.

It is clear that between the 2015–2019, 2017–2019 and 2019 data there is an increase in the number of things uploaded, as was expected from the data in table 2. Over and above that presented in the table, however, it can be seen that this a consistent trend throughout the year. Further, each dataset demonstrates a consistent relationship across the calendar year. This shows that summer—in the Northern Hemisphere—had fewer uploads than in the winter months for each of the datasets (approx. 10% difference).

From figure 2, it is evident the trend in things uploaded throughout the year was different between the baseline datasets and the COVID dataset. Between March and the end of June there is, time averaged over 21 days, a demonstrable increase in the actual number of uploaded things over the baselines. From then on, however, there is a reduction in the number of things uploaded, dipping below all three baseline data plots. Averaged over the course of the year, this meant the number of uploads in 2020 was 14 169 things greater than that for 2019. It appears that a shock event occurs around March that stems this change, which falls roughly inline with several Western Hemisphere COVID-19 case number increases [35,36]. This is represented on the figure through the vertical line for 15 March 2020.

**Figure 3.**
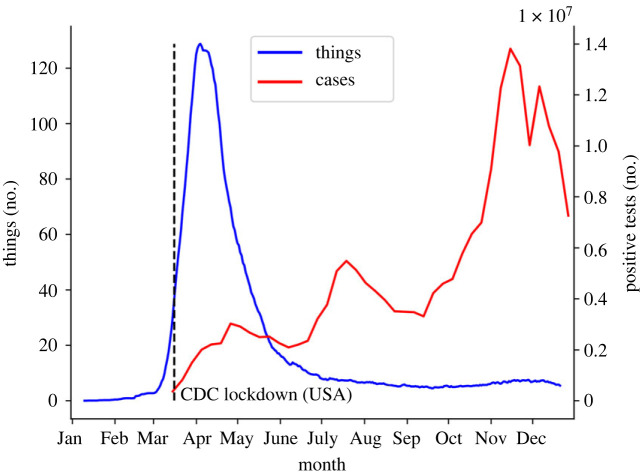
‘COVID’ things uploaded to Thingiverse in 2020 daily (averaged over 21 days). The case numbers were for the USA [36]. CDC, Centre for Disease Control.

To further investigate this relationship, the 21 day time-averaged daily uploads associated with the search term ‘COVID’ were plotted, as in figure 3. A total of 7053 things were found to have been uploaded in 2020 that relate to this search term, with a daily peak of 169 on 8 April 2020. This was equivalent to roughly 49.8% of the total increase in things uploaded compared to 2019. Also on figure 3 is a plot of the daily cases of COVID-19 in the USA (also time averaged to 21 days), provided by the Centre for Disease Control (CDC) [36].

From figure 3, it is possible to see roughly when the pandemic led to restrictions, at least in Europe, the USA and Canada that make up approximately 66% of the total ‘COVID’ uploads (that provided a recognized location). Further, it can be seen that there was a prolonged period of increased design effort from the Thingiverse community to produce designs that were related to the COVID-19 pandemic. From figure 3, it is further possible to see that the number of COVID things uploaded and the number of cases in several Western Hemisphere countries align well—at least for the initial rise in case numbers. Interestingly, however, there appears to be little relationship after the first peak. Further, the increase in the total number of things uploaded to Thingiverse over the whole of 2020 was much reduced compared to historical increases. As such, although the start of the COVID-19 pandemic appears to have led to an initial increase in design output, long term productivity appears to have decreased. This is reviewed in §6.

### 3.2. Types of thing

To develop an understanding of the type of things being uploaded to Thingiverse between 2015 and 2019 the tags assigned to things were analysed. The things uploaded in this period considered 257 598 unique tags with a total 3 368 997 tags used. This meant that each tag was used a mean approximately 13 times.

**Figure 4.**
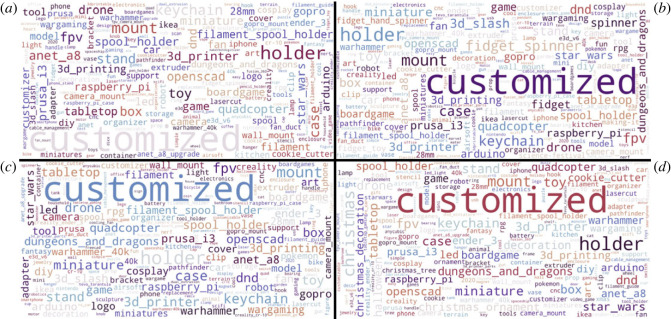
Word clouds for tags used between 2015 and 2019, broken down by quarter. (*a*) Q1, (*b*) Q2, (*c*) Q3, (*d*) Q4.

Using these tags, word clouds were generated, broken down by quarter, to observe annual trends—shown in figure 4. The top 10 tags for each quarter are also shown in table 3. It is demonstrated that the tags normally used by creators are varied and generally change very little throughout the year. ‘Customized’ was the most widely used word for all quarters—at an average of 124 000 per quarter (across all years). This aligns with a key benefit of three-dimensional printing being to modify products for specific use cases [37]. Similarly, the large range of alternate tags highlights the ability of the community to apply three-dimensional printing to a wide range of specialist problems and interests. Table 3 shows that customized is two orders of magnitude more common than the next most popular tag. Further the other nine tags are all the same order of magnitude. This trend is further exemplified when looking at the word cloud. Figure 4 shows that other descriptive tags not appearing in the top 10 appear with a similar frequency—without listing them all in a table. Additionally, ‘christmas’ and related tags are observable in the Q4 word cloud (figure 4*d*) but no others. This demonstrates that the uploaded things can, in some instances, follow real-world events and that these trends can be observed in the data. No other events are observable within these data, though it is recognized that this is unlikely given that the data are taken over several years.

**Table 3.** Top 10 tags for each quarter of the pre-COVID dataset (sum of Q1 2015–2019, Q2 2015–2019, etc.).

Q1		Q2		Q3		Q4	
customized	116 296	customized	126 293	customized	111 031	customized	142 385
holder	4262	holder	3931	holder	3630	holder	4045
mount	2872	mount	2700	mount	2478	christmas	3109
keychain	2362	fidget spinner	2143	case	1924	mount	2532
case	2144	keychain	2045	keychain	1852	fpv	1962
toy	2061	case	1984	fpv	1843	keychain	1961
stand	2003	fpv	1978	miniature	1756	toy	1918
fpv	1907	toy	1908	stand	1676	miniature	1903
box	1821	miniature	1750	toy	1647	stand	1881
anet a8	1819	3d slash	1712	dnd	1646	drone	1792

To understand whether and how the type of uploaded thing changed in 2020 owing to COVID-19, two datasets are considered. The first considers all 2020 uploads, as shown in table 4, while the second considers only the COVID uploads in 2020 (table 5). The 2020 dataset (table 4) shows that, at least for Q2, COVID related tags dominated the uploaded things. This aligns with that shown in figure 3, with Q2 demonstrating the peak COVID-related upload activity. As previously shown, however, after this initial spike in activity, there is little mention of COVID related tags in the remainder of the tag data, though Christmas related uploads can once again be recognized in the Q4 data. It would seem, then, that the maker community had grown tired of COVID related activity and was instead focusing on alternative designs—this notion is discussed further in §4. Also interesting was that ‘customized’ was missing from the Q4 2020 top 10 tags, the first time this was observed. It is currently unclear why this was the case.

**Table 4.** Top 10 tags for each quarter of the COVID (2020) dataset.

Q1		Q2		Q3		Q4	
customized	25 764	customized	51 520	customized	5638	holder	1338
holder	1260	COVID-19	3109	holder	1093	christmas	1153
dnd	1056	COVID19	1930	ender 3	858	ender 3	945
COVID-19	961	coronavirus	1663	mount	739	mount	828
ender 3	961	holder	1649	dnd	658	christmas ornament	672
coronavirus	933	mask	1273	miniature	621	stand	670
tabletop	859	ender 3	1247	tabletop	594	miniature	662
miniature	843	facemask	1186	stand	551	dnd	599
dungeons and dragons	820	coronavirus face mask	1075	warhammer 40k	550	case	592
mount	753	COVID	1017	case	533	creality	584

**Table 5.** Top 10 tags for each quarter for things tagged ‘COVID’.

Q1		Q2		Q3		Q4	
COVID-19	961	COVID-19	3109	COVID-19	380	COVID-19	381
coronavirus	626	COVID19	1648	COVID19	189	COVID	172
COVID19	451	coronavirus	1313	COVID	171	COVID19	158
mask	345	COVID	1017	mask	135	coronavirus	122
COVID	288	mask	841	coronavirus	122	mask	119
corona	166	facemask	804	facemask	110	facemask	89
facemask	124	coronavirus face mask	753	covidmask	108	covidmask	63
virus	122	covidmask	678	coronavirus face mask	105	christmas ornament	57
coronavirus defend	120	faceshield	516	surgical mask strap	59	coronavirus face mask	57
faceshield	117	COVID face shield	409	earsaver	53	christmas	49

Focusing on the ‘COVID’ tagged data more specifically through 2020 develops this understanding further (table 5). It can be seen that upload activity in Q3 and Q4 2020 for things tagged ‘COVID-19’—the top tag in all quarters for things related to COVID—was roughly 10% of that at the peak of activity in Q2 2020. This is despite significant increases in case numbers across these quarters (figure 3). Interestingly, 'christmas' remains a top tag in the Q4 2020 COVID thing tags while all other tags have been replaced with pandemic related words. This suggests that the two events (Christmas and COVID) have been combined to form designs within the maker community.

### 3.3. Community interaction of things

**Figure 5.**
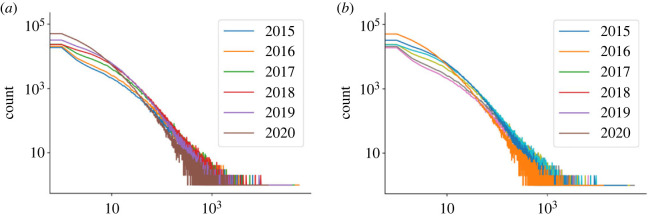
Log-log histograms of likes and comments on things between 2015 and 2020. (*a*) Likes and (*b*) comments.

Community interaction with things varies over time, as discussed in §2.2. This means that the number of likes and/or comments a thing has changes with time. Although the trend should be positive (i.e. the longer a thing has been published the more likes it will have, on average), greater insights could be drawn from analysis of the dynamics. Furthermore, it was thought necessary to better investigate the quality of data as issues were found with the number of likes presented (see §2.3). To do this, the number of likes and comments were analysed and plotted in figure 5*a*,*b* respectively.

Figure 5 shows that the number of likes and comments for uploaded things is often low for all years—with the median number of likes and comments being less than 2 for all years. Additionally, although the length of time things have been available for does seem to affect the number of likes and comments, this effect significantly reduces after the things have been available for approximately 3 years (for the data shown, uploaded in 2018 or earlier). There are several potential reasons for why things may not see increased interaction after being uploaded for 3 years, including:

(i) the thing is not promoted as actively on Thingiverse’s website owing to their own internal algorithms;
(ii) the things have been improved upon and new iterations are now available;
(iii) the problem that the thing was targeted at overcoming no longer exists; and
(iv) other products have become available on the open market that the maker community may now be able to purchase.

For more recent uploads, such as that in 2020, it is recognized that things would probably still be being actively liked and commented upon, and it should be expected that things from this period will have generally been interacted with less than for older uploads.

Investigation of things related to the search term ‘COVID’ in 2020 returned 5.8% of the total 2020 thing likes despite accounting for just 1.9% of all uploads. This was found to mean that each ‘COVID’ thing was liked approximately 37.9 times, compared to the average of approximately 12.6 for all things in 2020. Similarly, the number of comments each ‘COVID’ thing received was, on average, approximately 3.6 times higher than for the average thing uploaded to Thingiverse in 2020.

If the total dataset of things is looked at, dating back to 1 January 2015 and up to 31 December 2020, it is possible to see that, on average, the COVID things outperform the average thing (approx. 37.9 likes per thing compared to approx. 27.0 likes per thing). Additionally, and similarly to 2020 data, the number of comments per COVID thing was also higher (approx. 3.8 compared to approx. 1.5 comments per thing). These statistics are true despite COVID-related things being online for approximately 2 years less than the average thing in the dataset.

Demonstrably then, not only were there a significant number of COVID related things being uploaded in 2020, but the level of engagement between the ‘COVID’ things and community was higher than for the average thing—even when neglecting the temporal property of interaction.

The type of COVID thing that was being uploaded and interacted with could also be investigated. To do this, the COVID things with 500 or more likes from the dataset were found (which accounted for 75 things) with the tags used for these things analysed. The top 10 most common tags were (with the number of occurrences):

(i) ‘COVID-19’ (59);
(ii) ‘coronavirus’ (46);
(iii) ‘mask’ (35);
(iv) ‘COVID19’ (34);
(v) ‘COVID’ (26);
(vi) ‘facemask’ (24);
(vii) ‘corona’ (19);
(viii) ‘coronavirus_face_mask’ (18);
(ix) ‘covidmask’ (18); and
(x) ‘virus’ (15).

It could be observed that the most interacted with COVID things were generally those that related to face masks, ignoring the general COVID tags. This was thought likely to be as populations in Europe, the USA and Canada (the most active regions) became accustomed to wearing masks following government legislation. This is reiterated with four (ids 4249113, 4225667, 4635429 and 4616510) of the top five most liked COVID things related to making mask wearing either more comfortable or more effective. The fifth is an educational model of the SARS-COV-2 virus which was uploaded before the pandemic started. Other popular COVID things included face shields, door openers and decorations. Generally, then, the most popular designs that were related to ‘COVID’ were functional designs that either improved people’s protection against COVID-19 or made the experience of using this protection easier and/or more comfortable. It should be noted that it is popular to upload mask designs inspired by media to Thingiverse, although the authors do not expect this to have a significant impact on results.

Of the 10 most popular COVID things that were functional items on Thingiverse, only one was found to have been certified by a health body—a surgical ear strap (ID: 4249113) by Suraky [38], which was approved by the US National Institute for Health [39]. Interestingly, this was also the most liked COVID-related design on Thingiverse in 2020. However, it is important to note that popularity on Thingiverse, as measured by likes, does not inherently guarantee the use or exceptional quality of a design. While the ‘most liked’ designation can garner attention, it does not always correlate with practicality or suitability for its intended purpose. In this case, the surgical ear strap’s high likes were complemented by its certification, reinforcing its functional value. Comparatively, when contrasted with company and regulatory-led endeavours, the maker community appears to exhibit a comparatively lower reliance on producing approved and certified products. There are probably several reasons for this.

The first relates to reaction time of the community. For the masks and ear saver in the top 10 most liked COVID things, the latest upload date was 2 April 2020. In comparison, it took Prusa until 14 May 2020 to receive European Union certification for their face shield [28].

The second is that for certification every manufacturer must have individual certification if attempting to provide designs as PPE or medical devices [25,40,41]. This means that every maker would have to have an individual licence for the devices they manufacture. In practice, this is unlikely to be achievable for most of the maker community, with costs estimated by Prusa to be between 3000 and 5000 EUR [25]. In the UK, as an example, the government and Medicines and Healthcare products Regulatory Agency provided exemption criteria, though this still required approval [42] if described as PPE. If products were not being described as PPE or medical devices, it was possible for UK manufacturers to provide their products under the General Product Safety Regulations 2005 [42], though these should only have been used by the general public. This posed a significant barrier to entry for the maker community to help healthcare professionals while following the government guidelines.

## 4. Discussion

From the data collated and analysed within this work, there appears to be a correlation between the start of the COVID-19 pandemic and the activity on the Thingiverse design repository. This spans the number and type of things being uploaded, as well as how these things were interacted with, and was generally led by Western Hemisphere nations. The maker community moved fast and learned quickly, reaching a peak in activity within approximately one month of the CDC lockdown being brought in across the USA.

However, after an initial increase in design activity, reduction in design output from the community through 2020 was observed, despite COVID-19 cases still requiring restrictions and shut downs/lock downs. There will be many factors at play, and the data presented in this work cannot help us confidently identify which factors play a significant role. Further work investigating this would probably prove useful as it would enable efforts to motivate maker communities to be more effective. However, the authors speculate that the following may be some of the more significant factors, but reiterate that other factors will no doubt exist and could play a substantial role. The reasons listed are not in any particular order:

(i) scale up of matured supply chains in industry had caught up with the agility of the maker community, allowing larger quantities of product to be made more reliably and efficiently, potentially with better certification procedures;
(ii) reduced time at work, educational establishments and defined ‘maker-spaces’ led to reduced design output from the community as a whole, owing to lack of access to resource, prioritization of other tasks and reduced design opportunities encountered;
(iii) design fatigue started to set in, leading to the community taking a step-back from designing things after several months of significantly elevated output. A reduction in other real-world activities—for example, movie releases—limited the number of events/things to draw inspiration from, further drawing design fatigue;
(iv) the relaxation of restrictions in summer in many Western Hemisphere nations led to the community being able to return to work, see family and friends and generally access other activities that reduced design output;
(v) suitable designs had already been developed and shared for the individually identified problems that led to wide-spread fabrication of a limited number of designs;
(vi) as the pandemic wore on, and other world events occurred, the media coverage of local community efforts reduced. This meant that the maker community efforts were less well supported—by authorities, industry and the community itself;
(vii) maker communities may have shifted from general purpose repositories to store their designs (such as Thingiverse) to more specific or made for purpose ones as they were developed; and
(viii) finally, the reduction in output may have been from a pure reduction in demand, especially at a local level. This may, in part, have been owing to a deeper understanding of the virus being developed, allowing pragmatic approaches to changes in practice to be undertaken, such as reducing distancing rules and mask wearing in open environments.

## 5. Recommendations for supporting the maker community

The observed reduction in design output raises questions as to whether such an effect can be reduced or delayed, particularly for any future event. Although this is clear when dealing with shock events—such as the initial emergence of COVID-19 in the population—this may also provide benefit in more *normal* times; for example, allowing things to be designed to address humanitarian issues. The primary contribution of this paper has been in demonstrating the potentially significant impact of maker communities when tackling problems such as the spread of COVID-19. While not explicitly derived from the findings of this paper a secondary contribution is provided in the form of a set of policy recommendations. These are based on undertaking the research reported in this paper and the collective experience of the authors spanning both research and practice in maker-spaces and associated technologies, and participation in open source design projects [43]. This totals around 70 years of combined experience. The recommendations are:

(i) development of certification: it would be beneficial to the community to understand what regulation(s) needs to be met in the design and manufacture of parts for a given challenge, as well as to have a mechanism to achieve regulatory compliance for their things (as required). This is a challenge affecting many high-value industries adopting additive manufacturing into their workflows [44] and is further complicated through distributed design and manufacturing. At the other extreme, means of standardizng uploaded things—based on tag allocation or similar—would mean that design identification and grouping of things would be more streamlined;
(ii) allocate and promote funding: it has been shown that design output initially increased in response to COVID-19, much of this was from the free-will of the maker community, with few projects offering monetary incentives. Although this was able to stimulate a short-term increase in activity, long-term activity may have been hindered by the inability of the community to act within limited financial means—potentially worsened by the effect of the pandemic. To overcome this effect, funding opportunities for successful designs and/or involvement in projects may be required—similar to the journey of Linux [45]. It is not clear what effect this would have [46,47], nor what funding means would be required, at this time—future work may investigate this further. Allocation of funding to designs may be based on professional opinion or directly allocated through the community via interactions (likes, comments) and downloads/makes;
(iii) offer direction to improve motivation: as was previously highlighted, design-boredom may have affected the activity of the maker community after an initial period of increased activity. Developing challenges and activities that direct designers and the community more widely may enable this effect to be reduced. This should provide a range of different activities, with suitable breaks, to encourage creativity and reduce the risk of burnout, while balancing the broadness of design to allow creativity but without confusing the subject matter. Work investigating why makers contribute their own private resources (time, energy, filament, etc.) to public efforts has found intrinsic enjoyment and the expectation that ones own skills would develop as very effective [46–48]. Hence, the development of design challenges may help to increase designers enjoyment by game-ifying the design process, as well as provide metrics for designers to measure their skills increases while also directing the development of skills in the most relevant direction for the specific problem being addressed; and
(iv) develop avenues for recognition: lastly, it is thought that properly recognizing the contribution of individuals and the community more widely would be beneficial. This would provide motivation for the community, and would encourage collaboration within the community as individuals become more established and better known. Membership to a community has been shown to be an effective long-term motivator in open-source communities [46]. ‘Expected reputational benefits’ can actually help compensate for dips in motivation when the main motivator (enjoyment) is lacking.

Until this point, this work has used the quantity of designs produced (or things uploaded) as a positive metric of design performance. In the end, it is important to produce quality designs that gain acceptance throughout the community, as opposed to producing an abundance of designs that never see substantial use. Evaluating the quality of designs poses a challenge, and this is a question that surely extends beyond the realm of data presented here. However, it is true that with an increased design space exploration we are more likely to develop an improved or optimal product. Hence, design output could be correlated with improved design quality, but they are not identical metrics.

Finally, of the work presented here there remains an untouched area of significance. This is how the designs were actually used and how many of these designs proved useful in practice? Again, this is a challenging question to answer using the data collected during this work. However, some examples of products produced by the maker community in response to the pandemic and their applications can be provided. These are the production of test swabs by Formlabs [49] and the manufacture of face shields by Prusa [50]. The use of open source repositories enabled these face shields to be produced by groups around the globe [51].

## 6. Conclusion

The work within this paper has demonstrated the potential and direct value of the maker community by focusing on the response to the COVID-19 pandemic on the Thingiverse platform. It has been demonstrated that the community had a significant and direct response (3109 ‘COVID’ tagged things in Q2 2020) to the initial pandemic requirements and lockdown, before the response reduced in output (381 ‘COVID’ tagged things in Q4 2020). Additionally, it has been observed that the interactions with these designs (likes, comments) was significantly higher—both over the period of interest and otherwise—than for non-COVID things uploaded to the Thingiverse platform. However, it has not been possible to understand how these interactions changed with time—i.e. the dynamics of likes—owing to the restricted accessibility of data, meaning that only totals are available.

Analysis of the types of products being worked on revealed the most popular upload tags to be ‘mask’ (or similar such as ‘facemask’). This may have been owing to the effect of mass media—which started to heavily publicise the use of face masks at the peak of the maker community response. Notably, many of the most popular things uploaded continued to use similar tags to those used prior to the pandemic; this may be owing to retained popularity, but may also be owing to convergence and informal standardization of these tags within the particular communities.

It was found that the increase in design output relative to the length of the pandemic was short-lived. Design output reached a peak approximately one month after initial lockdowns were introduced to the USA, before dropping—approximately exponentially—for the following months. Several reasons for this have been hypothesized, related to real-world factors (such as industrial supply catching up to the agility of the maker community) but many are directly related to the *management* of the maker community’s response (text removed). For example, the spread in the use of tags is vast (over 2015–2019, 257 598 unique tags were used), and thus there is potential for valuable design output to be lost within the platform. Additionally, government and local authorities (whether this be on a regional, national or international scale) could have provided direction to improve the applicability of design output, e.g. through broad directives, direct use of the platform, or other marketing means. However, this would have reduced the openness of the response, which may have a negative effect on creativity. As such, a considered response should be taken based upon future needs.

Future work may also investigate what other events are observable in the Thingiverse data. This should be done to determine whether there are noticeable design activity changes for smaller events (such as movie releases, localized challenges, holidays, etc.). Alternatively, longer term studies might investigate how the community changes over time, both with regards to make-up and output, and what the community network may look like. These investigations would provide greater understanding around the maker community and its manifest behaviours and how the community can be best mobilized to help with design challenges in the future. Additionally, there is the potential for a higher level of classification and analysis of tags beyond exploring just the 10 most popular. For example, table 3 appears to have some keywords related to functional designs (stand, mount, case, and holder) and others related to novelty items (key chain, toy and miniature)—providing opportunity to organize tags semantically. Finally, it may be possible to draw additional insights through comparison of Thingiverse data with government datasets covering procurement of PPE.

## Data Availability

This paper presents aggregated anonymized data from Thingiverse. We are using data from the internet and made a particular effort not to use personal details. All files should be suitably licensed for use, and we have permission from the data holder (Thingiverse). The data needed to reproduce our results can be found on the Dryad data storage site with the DOI - https://doi.org/10.5061/dryad.gb5mkkwvr [34].

## Footnotes

1 The enabling of more non-designers to become involved in design [10].
